# Aesthetic emotions are affected by context: a psychometric network analysis

**DOI:** 10.1038/s41598-023-48219-w

**Published:** 2023-11-28

**Authors:** Yoed N. Kenett, Eileen R. Cardillo, Alexander P. Christensen, Anjan Chatterjee

**Affiliations:** 1 Faculty of Data and Decision Sciences, Technion – Israel Institute of Technology, 3200003 Haifa, Israel; 2 Penn Center for Neuroaesthetics, University of Pennsylvania, Philadelphia, PA USA; 3 Department of Psychology and Human Development, Peabody College, Vanderbilt University, Nashville, TN USA

**Keywords:** Human behaviour, Perception

## Abstract

Aesthetic emotions are defined as emotions arising when a person evaluates a stimulus for its aesthetic appeal. Whether these emotions are unique to aesthetic activities is debated. We address this debate by examining if recollections of different types of engaging activities entail different emotional profiles. A large sample of participants were asked to recall engaging aesthetic (N = 167), non-aesthetic (N = 160), or consumer (N = 172) activities. They rated the extent to which 75 candidate aesthetic emotions were evoked by these activities. We applied a computational psychometric network approach to represent and compare the space of these emotions across the three conditions. At the behavioral level, recalled aesthetic activities were rated as the least vivid but most intense compared to the two other conditions. At the network level, we found several quantitative differences across the three conditions, related to the typology, community (clusters) and core nodes (emotions) of these networks. Our results suggest that aesthetic and non-aesthetic activities evoke emotional spaces differently. Thus, we propose that aesthetic emotions are distributed differently in a multidimensional aesthetic space than for other engaging activities. Our results highlight the context-specificity of aesthetic emotions.

## Introduction

Aesthetic emotions (AE), long pondered by philosophers^1^, are also an object of empirical investigation^2^. While there is no agreed upon definition for aesthetic emotions, several candidate emotions have been proposed. AE are bound to intrinsic evaluation, in contrast to utilitarian emotions related to outcomes and goals^3^. Models of aesthetic processing typically incorporate emotions when a person engages with art^4–6^, literature^7^, and music^8,9^. Critically, most empirical research assumes that AE exist; whether they are a distinct type of emotions is disputed. The current study addresses this dispute by applying computational and empirical methods to characterize the AE space of recollections of aesthetic engagements as compared to other activities. We test the hypothesis that the emotional space of recalled aesthetically engaging activities is organized differently than in non-aesthetically engaging activities.

Menninghaus et al.^10^ claimed that AE constitute a special class of discrete emotions that explain variance in aesthetic perception and evaluation. By contrast, Skov and Nadal^11^ contend there are no AE and the beguiling concept be dropped entirely. Their opposing view is that the idea of AE is rooted in historical and philosophical traditions that have no psychological or neural support. In a spirited rebuttal, they claim empirical evidence fails to distinguish aesthetic from non-aesthetic emotions – for any purported AE (such as being moved). Rather, they contend that emotions in response to landscapes, art, design, or interior architecture, result from neurochemical processes that evolved to serve general biologically adaptive functions and appears conserved across species. They appeal to parsimony: distinct AE need not be evoked; ordinary emotions suffice. Nonetheless, the notion of AE continues to hold sway^6,12–17^.

In our approach, we adopt a constructivist framing of emotion (e.g.^18,19^). We agree with Skov and Nadal^11^ and Fingerhut and Prinz^20^ on the importance of grounding emotions in human biology, and more specifically to homeostatic states linked to arousal, valence, and approach-avoidance tendencies. Interoception underlying these homeostatic states consists of multidimensional, continuous, and fluctuating autonomic, visceral, endocrine, cardiac, and respiratory information. We also consider the roles played by language, categorization, and context in AE.

Most contemporary researchers agree that emotions have a physiological component^21^ with a phenomenological counterpart: We have a subjective impression of what it feels like to have emotions. We propose that a vocabulary for complex experiences^22^ enhances our sensitivity to distinct nuanced emotions. For example, we might distinguish between dejection, sorrow, and melancholy, or between awe, wonder, and delight. We anticipate that AE are affected by context. To be clear, we do not think that AE are a different species of emotions; we consider the possibility that they are a subset or combinations of emotions weighted differently in aesthetic experiences as compared to other emotion evoking activities.

### Measuring aesthetic emotions

Measuring or defining emotions is not straightforward, with current research viewing emotions as dynamic, highly variable and contextually defined^18,19,23–25^. Physiological markers of affect such as skin conductance, goose bumps, shivers, heart rate, and blood pressure can be measured^26–32^. Other physiological markers such as facial electromyographs can detect contractions of muscles associated with certain emotions, such as the zygomaticus when people smile, or the corrugator supercilii when people frown. Explicit observable behaviors like crying or laughing can also be informative^10^. These signals, while useful, can be difficult to interpret and typically lack the granularity afforded by language.

The most direct way to assess emotions, albeit imperfect, is to ask people to identify or label their feelings^33^. People are probably better able to recognize emotions than identify them, similar to how it is easier to recognize than to recall objects. We posit that using language, whether by generating terms or by matching words denoting emotions to experience can assess a person’s emotional state. Speakers within a culture adopt inexact but communal agreements to label, for example, interoceptive signals as wonder, horror, or joy. An emotion vocabulary brings specificity and nuance to our awareness of complex internal experiences and enhances our communicative capacity^22,34,35^.

Several tools attempt to characterize AE by asking participants about their experiences. Some assess basic emotions and others focus on specific domains of aesthetic experience, like music or consumer experiences^17^. Noting the need for a comprehensive assessment tool that encompasses the spectrum of potential AE, Schindler et al.^17^ developed the aesthetic emotions scale (AESTHEMOS). The AESTHEMOS includes both positive, mixed/negative emotions and relatively fine-grained distinctions. It includes commonly invoked AE (e.g. the feeling of beauty, fascination, or being moved), negative emotions (e.g. the feeling of ugliness, boredom, or confusion), emotions linked to pleasing and sense-making ways of enjoying aesthetics (e.g. humor, joy, vitality, and relaxation), as well as intellectual challenge, interest, and insight.

Research using the AESTHEMOS has applied computational methods to explore the conceptual space of AE^36–38^. For example, Hosoya et al.^38^ found that the space of aesthetics differentiated into three clusters: Negative emotions, positive emotions, and emotions indicative of appreciation, captivation, intellectual activity, and motivational states. The latter cluster are not purely pleasing but can be of mixed valence and complex. Beermann et al.^36^, using cluster analyses, identified 15 “families” of AE, and grouped them into four categories – negative, prototypical and often mixed, epistemic, and pleasing^36^. These studies did not survey the same emotions in other, non-aesthetically engaging, activities and are agnostic to our question: are AE evoked differently in aesthetic engagements than in other kinds of activities?

### The current study

We compared emotion response profiles across three types of recollections: aesthetic, non-aesthetic, and consumer activities. Participants were instructed to recall and report a specific memorable experience in detail (see Appendix 1). For the Aesthetic condition participants recalled a memory of being deeply engaged with fine arts (e.g. listening to music, watching a dance performance, looking at visual art, seeing a film, reading literature, looking at architecture). For the Non-Aesthetic condition, participants recalled being deeply engaged in activities that are not typically considered aesthetic (e.g. watching a debate, reading the news, listening to a lecture, witnessing an argument, etc.). For the Consumer condition, participants recalled a time when they were deeply engaged in buying something in person at a store. This condition offers an intermediate context between Aesthetic and non-Aesthetic conditions, in so far as it has an evaluative component like aesthetic activities but is instrumental in its aims unlike aesthetic activities. Thus, participants recalled activities that held their attention over an extended period of time but differed in the relevance of aesthetic qualities. For these recollections, we collected ratings on the list of 75 emotions (translated into English) from AESTHEMOS. Computational psychometric network analysis was conducted on these items across these three conditions, to represent and compare their emotional spaces.

Psychometric network analysis—based on mathematical graph theory—is suited to examining nuanced differences in complex patterns between conditions^39^. Network science has advanced the understanding of complex systems^40,41^ and is applied to represent language and memory^39,42–44^. Furthermore, the application of network science to analyze questionnaires—psychometric network analysis—has become common^39^ and is useful when analyzing multivariate data^39^. It complements existing techniques such as exploratory factor analysis and multidimensional scaling^45^. Importantly, psychometric network models provide a formal approach to bridge data analytical methods with theory^41^ and is suited for research on emotions^46^. Clusters derived from traditional dimensionality reduction approaches used in multivariate psychology research corresponds to clusters, or communities, identified formally over psychometric networks^45,47^. Beyond classifying items to clusters, psychometric networks convey additional meaningful information on the pattern of interaction of specific items and their organization, and is ideal for our question in this study.

## Methods

750 participants—250 participants per recall condition—were recruited online via Amazon mechanical turk (AMT)^48^. All participants were United States citizens whose native language was English. Participants were paid $4 for their participation, which took 30 min. This study was approved by the University of Pennsylvania Institutional Review Board. To address potential issues with AMT data quality^49^, we manually scrutinized the raw narrative responses data (see below). Participants were excluded from final data analysis if they: (1) entered non-sensical or irrelevant responses; (2) did not comply with instructions (for example, reporting an aesthetic engaging activity in the non-aesthetic engaging condition); (3) copy-pasted responses into the text box potentially indicating they are bots; (4) had poor English syntax suggesting they are not native English speakers; and (5) provided non-specific or general activity narratives. In addition, participants who did not complete the AESTHEMOS were excluded. These stringent criteria led to an exclusion of about 36% of participants. The final sample included 499 participants overall across the three conditions (see Table 1 for demographic information of the three groups).

**Table 1:** Demographic information for the three aesthetic engagement conditions.

	Aesthetic	Non-aesthetic	Consumer
N	167	160	172
M/F	85/82	86/74	97/80
Age	36.0 (11)	34.9 (10)	35.6 (11)
Education	15 (2.8)	14.9 (1.8)	14.9 (2.1)
Ethnicity W, AA, HL, A, OP	83%, 8%, 4%, 2%, 2%	78%, 6%, 6%, 9%, 1%	83%, 11%, 2%, 3%, 1%

*M/F* number of male/female participants, *Age* average age in years (SD in parentheses), *Education* average years of education (SD in parentheses), Ethnicity: *W* White, *AA* Aferican American, *HL* Hispanic/Latino, *A* Asian, *O* other/Preferred not to say.

## Materials

### Recall immersive activity task

Participants were randomly assigned into one of three conditions: Aesthetic Engagement, Non-Aesthetic Engagement, and Consumer-Aesthetic Engagement. The recall immersive activity task was similar across the three conditions, requiring the participant to recollect and report an immersive activity that was either related to an arts experience, a non-aesthetic experience, or a consumer-related experience (see Appendix 1 for instructions). Aesthetic experiences were defined by examples of engaging with the arts—reading a novel, watching a film, listening to music, etc. Non-aesthetic experiences were defined as reading, watching, listening to etc.; that is, activities where aesthetics is not of central relevance (e.g. the news, a political debate, a lecture). Consumer experiences were defined as buying a book, a phone, clothing, etc. Participants were required to type their recollected memory, with at least 500 words. After reporting their specific memory and rating the AESTHEMOS emotions attributed to their memory (Appendix 2), participants answered questions about the quality of their recollected memory and potential domain expertise confounds. Specifically, participants were asked to report the following aspects of their recollection: (1) How difficult was it to retrieve the memory? (2) How vivid was their memory of this experience? And (3) How intense were their feelings during this experience, and (4) How long ago did it occur? To control for potential domain expertise confounds related to their specific aesthetic conditions, participants were asked to answer whether they have professional expertise or formal training related to their specific recalled experience and to the general domain (e.g. arts, consumer, etc.).

### The aesthetic emotions scale

The aesthetic emotions scale (AESTHEMOS)^17^ was developed by converging various aesthetic emotions assessment scales (covering aesthetic domains such as art and music) to a list of 75 items, which were further reduced to a shorter 42-item instrument. It is postulated to apply to any of the arts and to other experiences, such as viewing nature, appraising design, or selecting consumer products.

To maximize comparison across contexts, we administered the full set of 75 items to our participants. The scale was constructed in German and also translated into English^17^. Participants read the items exactly as translated to English in Schindler et al.^17^, but for ease of visual representation in the network figures, we shortened each item to its adjective form. For example, “It filled me with longing” was abbreviated to *longing* or “Made me nostalgic” was reduced to *nostalgic* (Appendix 3).

### Psychometric network analysis

The AESTHEMOS networks of the three conditions were represented using a Psychometric network approach^50–53^. In these networks, nodes represent the 75 different AESTHEMOS items and edges represent endorsement associations between items—the tendency of the sample to endorse item b, given that item a is endorsed.

To estimate edges in the networks, we computed cosine similarity across all items to construct a 75 × 75 AESTHEMOS adjacency matrix (i.e. associations between each item endorsement) for each group^54^. Cosine similarity is an established method for calculating the angle of two vectors in an abstracted space and is commonly adopted in latent semantic analysis of text corpora^55^. Cosine similarity defines the co-occurrence probability of two words abstracted as normalized vectors. Ranging from 0 to 1, a cosine similarity of 1 represents two items that always co-occur, while a value of 0 represents two items that never co-occur.

To minimize spurious edges, the Triangulated Maximal Filtered Graph TMFG^56^ approach was used. The TMFG approach constructs a subnetwork, from the endorsement association matrix, that captures the most relevant information between nodes that are embedded in the original network and minimizes spurious associations. The resulting subnetwork is a clique-tree composed of four-node cliques connected with three-node cliques, and it retains a total of 3n–6 edges from the original network. The TMFG method begins by sorting all edge weights in descending order and adding the largest edge weight one by one, on the basis of an iterative construction process of a topologically constrained network (i.e., planar). In this construction, the algorithm adds a node into three-cliques, on the basis of a T_2_ move^56,57^. The T_2_ move inserts a node into any three-clique’s center where edges are added to it, forming a tetrahedron and keeping the network planar. When adding these nodes, the algorithm optimizes a score function that ensures the added node has the maximum increase in the sum of the additional edge weights. This approach retains the same number of edges between the conditions and avoids the confound of different network structures arising from different number of edges^52,58^. Thus, the networks constructed by this approach can be compared directly because they have the same number of nodes and edges. To examine the structure of the networks, the edges are binarized so that all edges are converted to a uniform weight (i.e., 1). Although the networks could be analyzed using weighted edges (weights equivalent to the similarity strength), this potentially adds noise to the interpretation of the structure of the network. Thus, the networks are analyzed as unweighted (all weights are treated as equal) and undirected (bidirectional relations between nodes) networks.

### Network visualization

The three AESTHEMOS networks were visualized using the Cytoscape software^59^ via the force-directed layout^60^. A force-directed layout minimizes overlap in network visualization, evenly distributes nodes and edges, and organizes nodes (AESTHEMOS items) such that edges are of a similar length. Such a visualization maximizes network visibility and comparability. In these 2D visualizations, nodes (AESTHEMOS items) are represented as circles and links between them are represented by lines. Since these networks are unweighted and undirected, the links convey symmetrical relations between two nodes.

### Network analysis

Networks can be analyzed across three different levels of analysis: macro-, meso-, and micro-levels. At the macro, global level of analysis, general measures of the entire topology of the network are examined. The meso, community level of analysis, addresses how the structure of the network partitions into clusters. The micro, node level of analysis, examines the specific significance of nodes. All three levels of analysis can convey meaningful cognitive information^43,44^. Network analyses were performed with the Brain Connectivity Toolbox^61^ for Matlab (specific scripts used will be described in parantheses), as well as standard Matlab functions. The Brain Connectivity Tollbox is a popluar toolbox in analyzing networks in general, such as brain networks or cognitive networks^61^. For future replication and extension of this work, a similar analysis can be conducted in R using the Semantic Network Analysis pipeline^62^.

To examine our hypothesis, we conduct several types of network analyses across the three levels. First, we quantify and compare macro-level network properties of the three networks. Next, we examine how items in each of the networks cluster together, and how these clusters correspond across the three networks and correspond to previous studies using the AESTHEMOS^17,38,63^. Finally, we identify the emotions comprising the core of the network, to examine the similarity of emotions constituting the core of each engagement condition. Furthermore, we examine any differences in the organization of emotion items across the three AESTHEMOS networks^64,65^. We do so by comparing the similarity of the three networks at the global, macroscopic level (structure similarity) and the intermediate, mesoscopic level (community similarity). Such analyses allow us to quantify the similarity of the emotional space related to the three conditions.

#### Macro-level network analysis

At the macro-level, the clustering coefficient (CC; measuring network connectivity, clustering_coef_bu.mat), average shortest path length (ASPL; measuring global distances, charpath.m), and network global modularity (Q; measures overall clustering of the network, modularit_und.m) were calculated^43^.

CC refers to the extent that two neighbors of a node will themselves be neighbors (i.e., a neighbor is a node *i* that is connected through an edge to node *j*), averaged across all nodes in the network. A higher CC relates to greater overall connectivity in the network. In semantic memory networks, such connectivity denotes the similarity between concepts (nodes) and has been related to creativity^66^. In our study, higher CC would indicate that emotions were similarly experienced as a result of a recalled activity.

ASPL refers to the average shortest number of steps (i.e. edges) needed to traverse between any pair of nodes, e.g. the higher the ASPL, the more spread out a network is. Previous research in semantic memory networks has shown that ASPL between concepts (nodes) corresponds to participants judgments of how closely two concepts (nodes) are related^67,68^. In our study, higher ASPL would indicate that emotions experienced to a recalled activity are more specified and “separated” from each other.

Q estimates how a network partitions into smaller sub-networks or communities^69,70^. It measures the extent to which a network has dense connections between nodes within a community and sparse (or few) connections between nodes across different communities. The higher Q is, the more the network sorts into subcommunities. Network communities correspond to traditional approaches of dimension reduction^45,71^, and convey meaningful information in aesthetic research^22,47,64^. In our study, higher Q would indicate that emotions experienced to a recalled activity cluster more to sub-classes (or categories) of emotions.

The group-based network analysis computes a single value for each network measure for the different networks (CC, ASPL, and Q). In order to statistically compare the three networks, we applied a bootstrap method^72^ to simulate a large distribution of the network measures from the empirical data and compare partial networks for each of the conditions. The bootstrapping procedure involves a random selection of half of the nodes comprising the networks (37 nodes). Partial networks were constructed for each AESTHEMOS network separately for these selected nodes. This method is known as the without replacement bootstrap method^73^. Finally, for each partial network, the CC, ASPL, and the Q measures were computed. This procedure was simulated with 1000 realizations. The difference between the bootstrapped partial networks on each network measure was then tested using a one-way ANOVA and simple effect analyses were conducted via independent samples *t*-test analyses.

To assess the similarity of the Aesthetic, Non-Aesthetic, and Consumer network structures, the Jensen-Shannon distance (JSD) measure was used. JSD is an entropy-based function that compares the spectral properties of two networks. The spectral properties of a network contain information about the connectivity between nodes and community structure^74^. The JSD was computed in R via EGAnet^75^. The JSD is a relative measure and therefore only provides numeric comparison rather than statistical comparison. Recent work has shown that JSD can be used to cluster networks into groups^76,77^. Further, a non-spectral-based JSD variant is the basis for network comparison of Bayesian Gaussian Graphical Models in the psychometric network literature^78^. Following the notation from De Domenico et al.^76^, JSD is defined as:

$${h}_{A}= -\mathrm{Tr}\left[{L}_{G}{\mathrm{log}}_{2}{L}_{G}\right],$$

where ${L}_{G}=c\times \left(D-A\right)$ is the combinatorial Laplacian rescaled by $c$ or one over the sum of the weights in the network. $A$ is the network and $D$ is a matrix with the sum of each node on its respective diagonal. ${L}_{G}$ is a density matrix used to compute Von Neumann entropy:

$${h}_{A}= -\sum_{i=1}^{N}{\lambda }_{i}{\mathrm{log}}_{2}\left({\lambda }_{i}\right),$$

where $\lambda$ is the eigenvalues of ${L}_{G}$. With the Von Neumann entropy, the Jensen-Shannon Divergence can be computed by computing the Kullback–Leibler Divergence of the two networks and their combined network:

$${D}_{JS}(\rho |\left|\sigma \right)=h\left(\mu \right)-\frac{1}{2}\left[h\left(\rho \right)+h\left(\sigma \right)\right],$$

where $\rho$ and $\sigma$ are ${L}_{G}$ of each network being compared and $\mu =\frac{1}{2}\left(\rho +\sigma \right).$ Taking the square root of ${D}_{JS}$ produces a bound metric [0, 1] that is referred to Jensen-Shannon Distance. Because JSD is a distance, smaller values going toward zero are an indication of greater similarity; larger values going toward one are an indication of lower similarity.

#### Meso-scale network analysis

At the meso-level, we conducted community detection analyses to examine how well the emotion items (nodes) in the networks cluster into well-defined categories. To do so, we apply a Matlab-based data driven approach to determine community assignment of each node in all three networks^79^. We applied a modularity maximization approach that aims to partition a network into communities. This approach uses the Louvain modularity method, a greedy stochastic method^80^. Given the stochasticity of this method, the application of the Louvain modularity method is reiterated 1000 times^81^. To resolve the variability across the 1000 iterations of the community assignment partitions, a consensus analysis is conducted to identify the community assignment partition that summarizes the commonalities across the entire distribution of partitions^79,82^. The results of this process are data-driven consensus-based identified communities for each of the networks and community assignment of each of the items (nodes) in the network to a specific community. Previous psychometric network research has demonstrated how such communities correspond to clusters identified in dimension reduction approaches, and can thus allow us to further examine the organization of AESTHEMOS items into clusters across our three conditions, compared to previous research^63^. Then, we computed the Rand Similarity Index between the three networks^83^, using the network community toolbox in Matlab (http://commdetect.weebly.com). The Rand similarity index measures the similarity between two partitions, corresponding to the fraction of node pairs identified the same way by both partitions (either together in both or separate in both partitions). Therefore, we computed all pair-wise Rand index scores of the three emotion networks.

#### Micro-level network analysis

At the micro-level, we compute the core/periphery role of each node in the network. The core-periphery analysis was conducted via the Brain Connectivity Toolbox in Matlab^61^ (core_periphery_dir.m). The core-periphery structure of a network has been found across different domains to be a critical network property^84–86^. It refers to nodes in the network being in either one of two qualitatively distinct categories: a dense “core” of tightly connected nodes and a sparse “periphery” of nodes loosely connected to the core and among each other^84^. In our task, core nodes would reflect emotions consistently rated as highly related to the recalled activity; whereas peripheral nodes would reflect emotions generally rated low or with greater variability across participants.

We apply a classic “two-block model” core-periphery analysis proposed by Borgatti and Evans^85^. This model proposes that nodes in a network are arranged into two groups, the core and the periphery, such that “core nodes are adjacent to other core nodes and some periphery nodes, while periphery nodes do not connect with other periphery nodes”^85^. Such an analysis allows us to examine the core emotions across the three networks – emotions that participants as a group recalled most consistently across conditions.

### Procedure

Participants signed an informed consent form and then completed all tasks using Qualtrics. Participants first completed the recall immersive activity task. After being presented with the instructions (Appendix 1), participants wrote their narratives in a text box. Participants could not complete this task before entering at least 500 words. After describing their recalled activity, they were asked four questions about the quality of the memory (time since the event, difficulty of its recall, and its vividness and intensity) and to rate their expertise and familiarity with the arts and with the domain of the reported memory. Next, they completed the AESTHEMOS related to the specific activity they reported (Appendix 2). AESTHEMOS items were presented one at a time and participants rated them on a 5-point Likert scale how much a specific AESTHEMOS item related to their narrative (1 = not at all; 5 = very much; Appendix 3). Participants answered several demographic questions. All methods in this study were conducted in accordance with APA guidelines for the ethical treatment of human participants.

## Results

**Figure 1:**
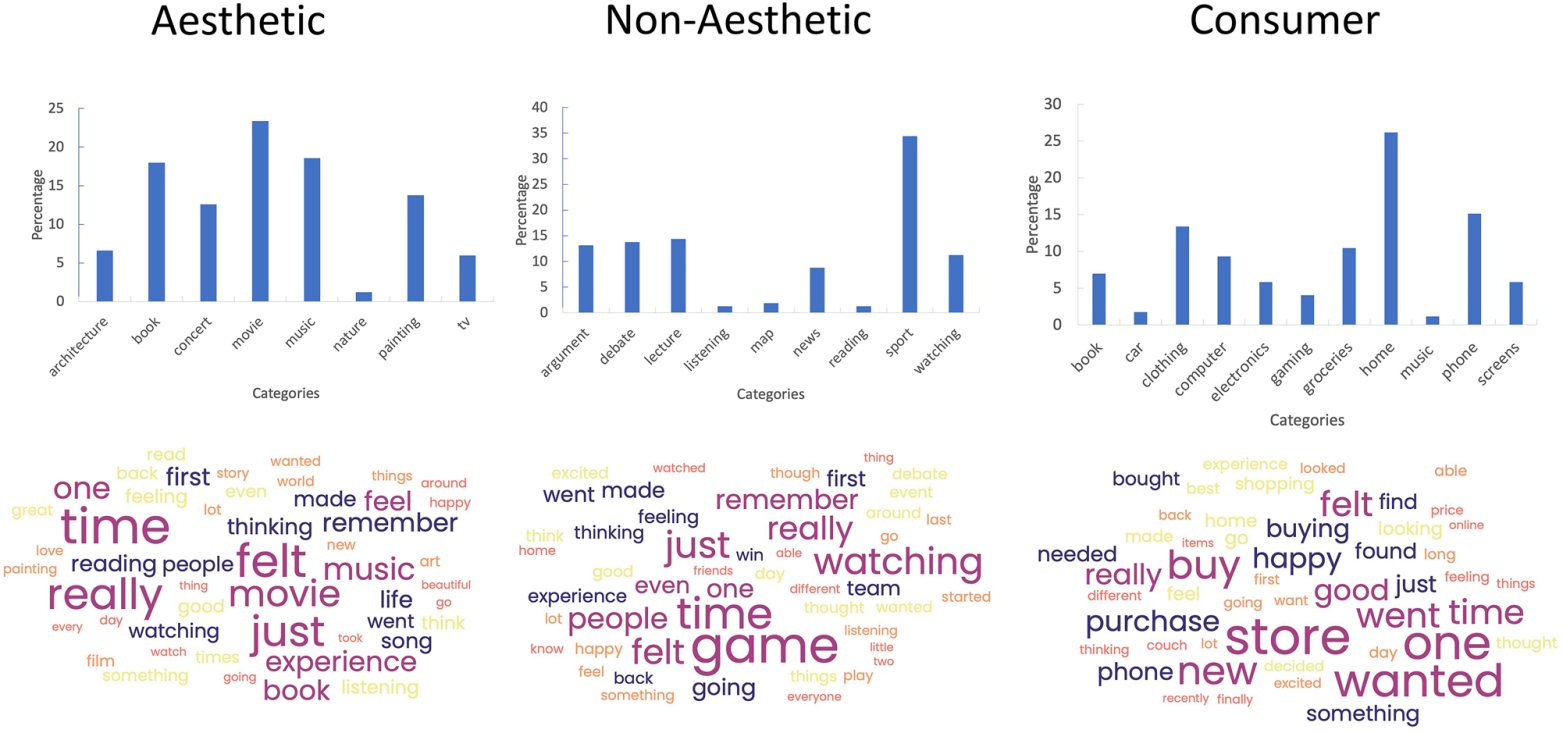
Qualitative analysis of narratives in the aesthetic immersion task. *Top*: classification of narratives to different categories. X-axes – different content categories; Y-axes – frequency of narratives. *Bottom*: Word clouds of the top fifty terms across all narratives in each condition. The larger the size of the term, the higher its frequency across the narratives. In the Aesthetic condition, participants’ narratives related to architecture, books/reading, concerts, movies, music, painting, and watching TV. The most frequent reported category were movies, reading, and music. The key terms included experiencing and feeling. In the Non-Aesthetic condition, participants’ narratives related to arguments, books, cooking, discussions, hearing lectures, listening, looking at maps, politics, reading, sports, and general watching activities. The most frequent reported category was sports, politics, and general watching. The key terms included thinking, remembering, and going. In the Consumer condition, participants’ narratives related to purchasing house goods, clothes, and phones. The key terms included buying, wanting, and feeling.

For an initial qualitative content analysis of participants' narratives, we categorized their reported experiences. We recognize that the boundaries between what we call Aesthetic, Non-Aesthetic, and Consumer activities may be indistinct. For example, one can look at an artwork and evaluate how much it might cost and impress friends. One could look at a teapot and admire its shape and glaze without an acquisitive desire. One could look at a sporting event and admire the beauty of movement and the elegance of well-toned bodies. As a check that these encounters were treated differently, we entered the entire narratives into a word-cloud analysis (https://www.freewordcloudgenerator.com/generatewordcloud) to identify the top fifty terms generated in each engagement condition (Fig. 1). This qualitative analysis confirmed that participants differentiated activities and experiences across the three conditions, using overlapping and distinct terms in their narratives.

There were no differences in the difficulty of recalling the different events, or in any domains of expertise (all *p*’s > 0.05). However, vividness and intensity of the recalled memory differed. A condition (Aesthetic, Non-Aesthetic, Consumer) one-way ANOVA revealed a main effect of Condition on vividness, *F*(2, 496) = 3.69, *p* = 0.025, $\eta^{2}$ = 0.015. Post-hoc independent-samples *t*-test analyses revealed that Aesthetic memories (M = 85%, SD = 17%) were less vivid than those of the Non-Aesthetic (M = 89%, SD = 12%), *t*(337) = − 2.46, *p* = 0.015, d = 0.27; and the Consumer condition (M = 89%, SD = 14%), *t*(337) = − 2.1, *p* = 0.036, d = 0.26. No differences were found in vividness between the Non-Aesthetic and Consumer conditions (*p* > 0.05).

A condition (Aesthetic, Non-Aesthetic, Consumer) one-way ANOVA revealed a main effect of Condition on emotional intensity, *F*(2, 496) = 19.39, *p* < 0.001, $\eta^{2}$ = 0.073. Post-hoc independent-samples *t*-test analyses revealed that Aesthetic  memories (M = 82%, SD = 17%) were not more intense than Non-Aesthetic memories (M = 80%, SD = 18%), *t*(337) = 1.31, *p* = 0.192, d = 0.11; and more intense than those of the Consumer condition (M = 70%, SD = 23%), *t*(337) = 5.67, *p* < 0.001, d = 0.59. Finally, Non-Aesthetic memories were more intense than Consumer memories, *t*(337) = 4.35, *p* < 0.001, d = 0.48. Thus, while Aesthetic memories were the less vivid across conditions, they were recalled most intensely.

### Macro-scale network analysis

**Figure 2:**
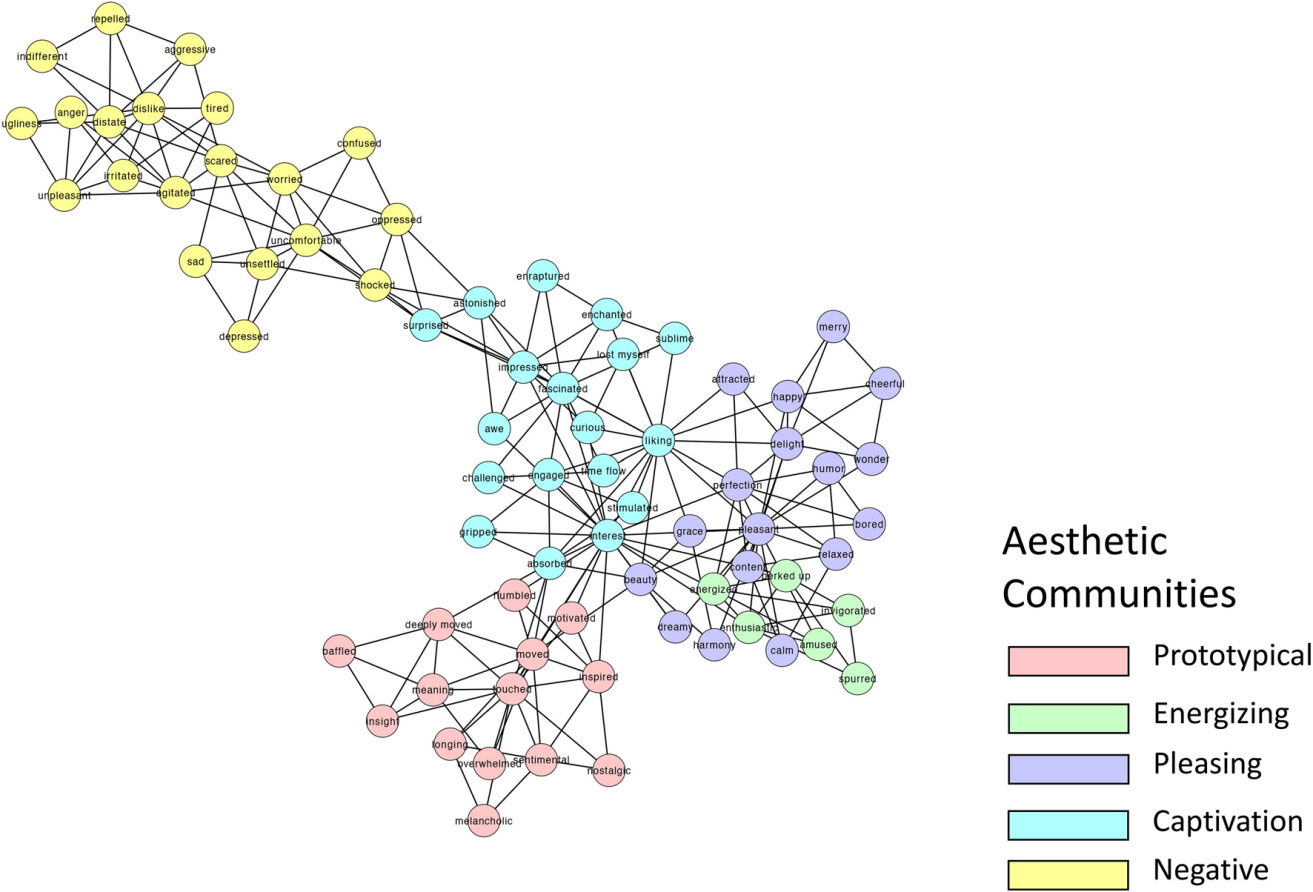
2D visualization of the Aesthetic AESTHEMOS network of the 75 AESTHEMOS items (nodes). Edges denote symmetrical relations between nodes. Color of nodes represent their data-driven derived community assignments.

**Figure 3:**
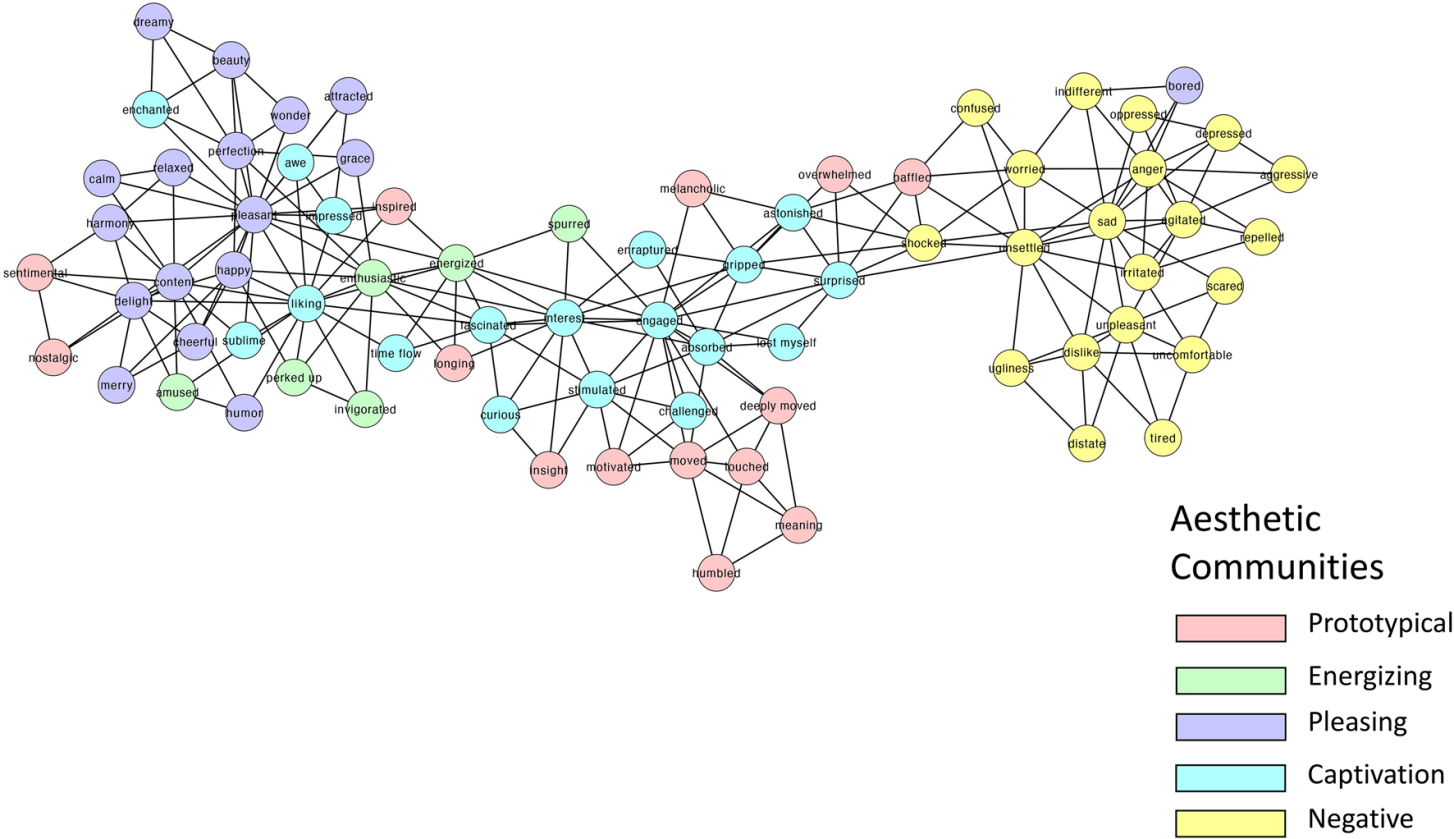
2D visualization of the Non-Aesthetic AESTHEMOS network of the 75 AESTHEMOS items (nodes). Edges denote symmetrical relations between nodes. Color of nodes represent their data-driven derived community assignments, based on the Aesthetics network community partition.

**Figure 4:**
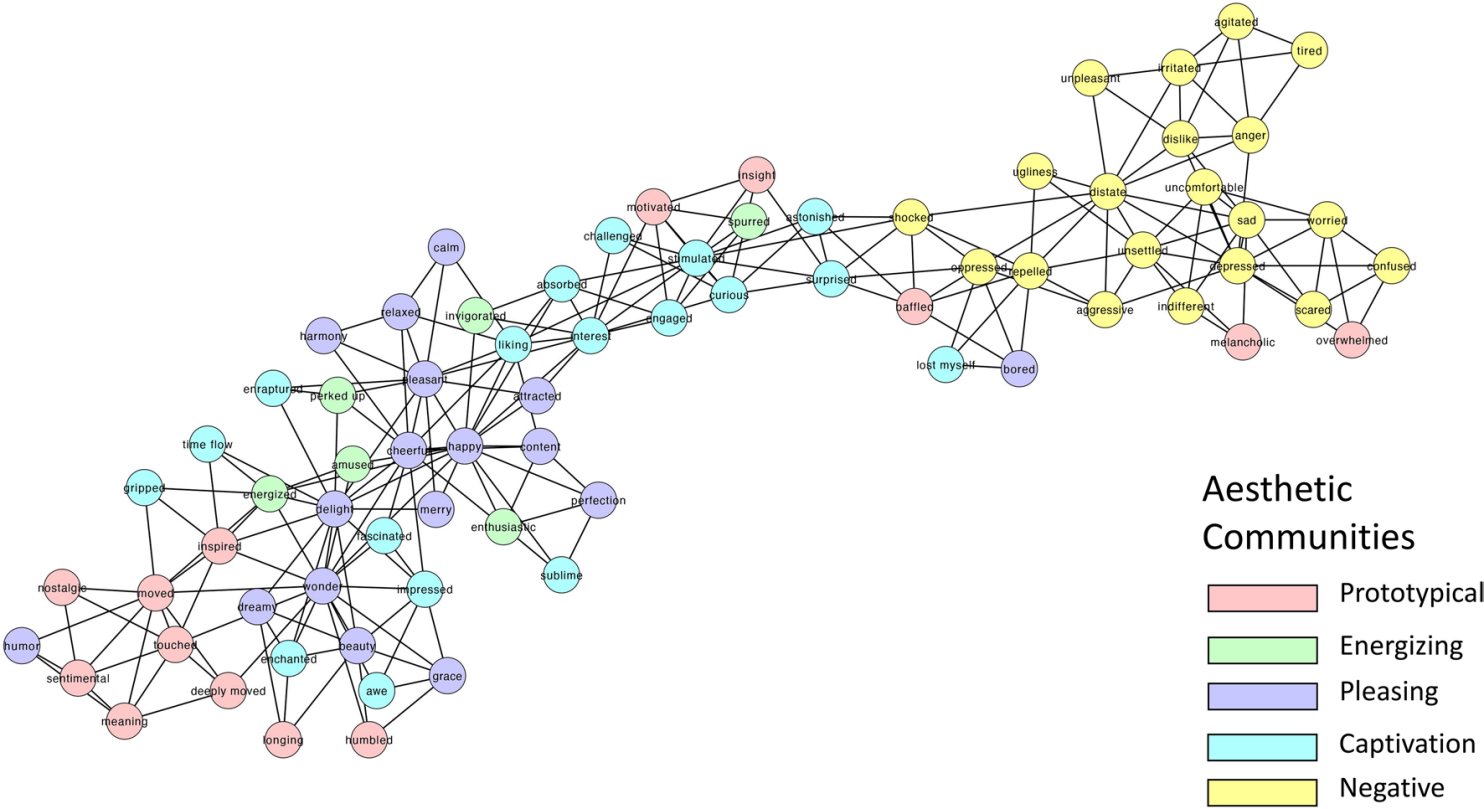
2D visualization of the Consumer AESTHEMOS network of the 75 AESTHEMOS items (nodes). Edges denote symmetrical relations between nodes. Color of nodes represent their data-driven derived community assignments, based on the Aesthetics network community partition.

**Figure 5:**
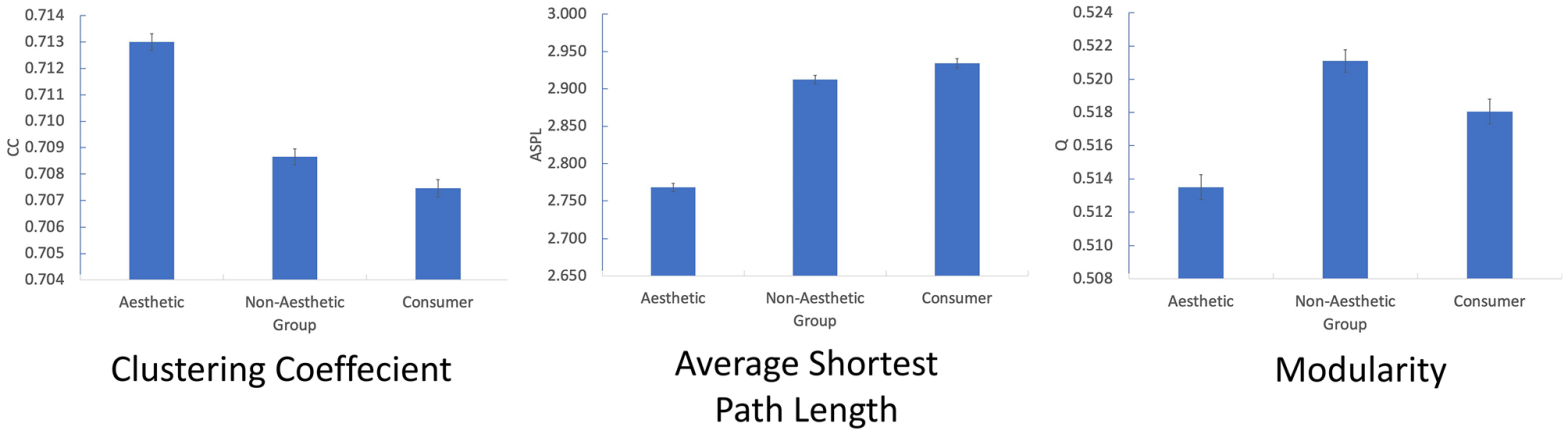
Partial network analysis for CC (left), ASPL (center), and Q (right). X-axis – Aesthetic, Non-Aesthetic, and Consumer conditions. Y-axis – dependent variables (CC, ASPL, and Q; Error bars denote standard error).

Next, we represented the Aesthetic (Fig. 2), Non-Aesthetic (Fig. 3) and Consumer networks (Fig. 4). The group-based network analysis computes a single value for each network measure for the different networks (CC, ASPL, Q). To test the statistical significance of differences between the networks, we applied a bootstrapped partial networks analysis^73,87^ and generated a distribution of values (Fig. 5).

*CC:* A condition (Aesthetic, Non-Aesthetic, Consumer) one-way ANOVA revealed a main effect of Condition on CC, *F*(2, 2997) = 88.61, *p* < 0.001, $\eta^{2}$ = 0.056. Post-hoc independent-samples *t*-test analyses revealed that the CC of the Aesthetic condition (M = 0.713, SD = 0.01) was larger than that of the Non-Aesthetic condition (M = 0.708, SD = 0.01), *t*(1998) = 10.18, *p* < 0.001, d = 0.5; and larger than that of the Consumer condition (M = 0.707, SD = 0.01), *t*(1998) = 12.39, *p* < 0.001, d = 0.6. Furthermore, the CC of the Non-Aesthetic condition was larger than that of the Consumer condition, *t*(1998) = 2.72, *p* = 0.003, d = 0.1.

*ASPL:* A Condition (Aesthetic, Non-Aesthetic, Consumer) one-way ANOVA revealed a main effect of Condition on ASPL, *F*(2, 2997) = 234.902, *p* < 0.001, $\eta^{2}$ = 0.136. Post-hoc independent-samples *t*-test analyses revealed that the ASPL of the Aesthetic condition (M = 2.768, SD = 0.18) was smaller in the Non-Aesthetic condition (M = 2.912, SD = 0.18), *t*(1998) = -18.06, *p* < 0.001, d = 0.8; and smaller than that of the Consumer condition (M = 2.934, SD = 0.20), *t*(1998) = -19.85, *p* < 0.001, d = 0.87. Furthermore, the ASPL of the Non-Aesthetic condition was smaller than that of the Consumer condition, *t*(1998) = -2.52, *p* = 0.006, d = 0.12.

*Q:* A condition (Aesthetic, Non-Aesthetic, Consumer) one-way ANOVA revealed a main effect of Condition on Q, *F*(2, 2997) = 28.063, *p* < 0.001, $\eta^{2}$ = 0.018. Post-hoc independent-samples *t*-test analyses revealed that the Q of the Aesthetic condition (M = 0.513, SD = 0.02) was smaller than the Non-Aesthetic condition (M = 0.521, SD = 0.02), *t*(1998) = − 7.63, *p* < 0.001, d = 0.4; and smaller than the Consumer condition (M = 0.518, SD = 0.02), *t*(1998) = − 4.31, *p* < 0.001, d = 0.25. Furthermore, the Q of the Non-Aesthetic condition was larger than that of the Consumer condition, *t*(1998) = 3.02, *p* < 0.001, d = 0.15.

Finally, we compared the global similarity of the networks via the Jensen-Shannon Distance (JSD). JSD scores range between 0 (identical structures) to 1 (completely different structures)^77^. Our analysis revealed that the JSD score between the Aesthetic and Non-Aesthetic networks was 0.291, between the Aesthetic and Consumer networks 0.322, and between the Non-Aesthetic and Consumer networks was 0.317.

Overall, the Aesthetic network was the most flexible^66^: It had the highest overall connectivity (CC), with shortest distances between nodes (ASPL), and was the least organized into subcategories (Q). Furthermore, at this level of analysis, the Aesthetic network was most similar to the Non-Aesthetic network and the Aesthetic and Consumer networks were the least similar.

### Meso-scale network analysis

Next, we identified communities across the three networks. This analysis led to five communities for the Aesthetic network, six communities for the non-aesthetic network and three communities for the Consumer network (Appendix 3). Since our focus is on the Aesthetic condition, we examine the distribution of the Aesthetic communities across the two other networks (Figs. 3 and 4). We labeled the communities based on the nodes within each cluster and informed by past research as: (1) Prototypical; (2) Energizing; (3) Pleasing; (4) Negative; and (5) Captivation.

**Figure 6:**
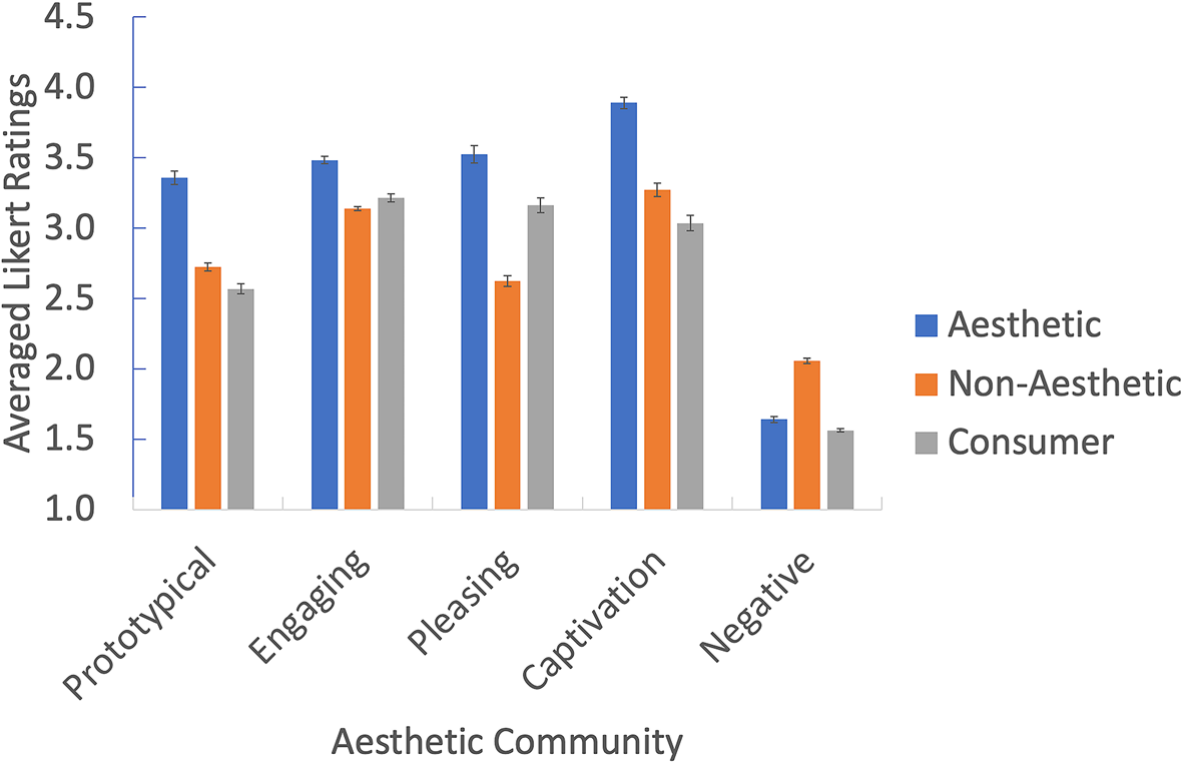
Averaged Likert scores of the relatedness of AESTHEMOS item communities across the three conditions. Communities are based on the Aesthetic network community analysis.

Examining the averaged Likert scale ratings for each of the items comprising these communities across the three conditions (Fig. 6) using a Community x Condition between-sample ANOVA revealed a main effect of Condition, *F*(2, 210) = 15.659, *p* < 0.001, $\eta^{2}$ = 0.13 a main effect of Community, *F*(4, 210) = 89.280, *p* < 0.001, $\eta^{2}$ = 0.63, and a Condition x Community interaction effect, *F*(8, 210) = 5.908, *p* < 0.001, $\eta^{2}$ = 0.18.

Finally, the similarity across communities of the three networks were computed using the Rand index between the community partitions^83^. Higher Rand scores indicate more similarity between two partitions. We found that the Rand index between the Aesthetic and Non-Aesthetic partitions was 0.778, between the Aesthetic and Consumer partitions was 0.801, and between the Non-Aesthetic and Consumer partitions was 0.748.

Overall, the Aesthetic network consists of five communities, which largely accord with previous research on aesthetic emotions. Notably, activities in Aesthetic condition were remembered with higher ratings of prototypical, engaging, pleasing, and captivating emotions than the other two conditions, consistent with the reports of greater intensity of their recall. In all three conditions, negative emotions were consistently not prominent in their recollections. Finally, at this level of analysis, the community structure of the Aesthetic and Consumer networks were most similar, and the Non-Aesthetic and Consumer networks the least similar.

### Micro-scale network analysis

Next, we examine the similarity of the networks at the local, microscale level, by conducting a core-periphery analysis^85^. We found that 31 nodes comprise the Aesthetic network core, 26 for the Non-Aesthetic network, and 30 nodes for the Consumer network (Appendix 3). We qualitatively examine unique core emotions for each context as well as the shared core emotions across all contexts (unique vs. specific core emotions). We find 13 shared core emotions across all contexts: *moved*, *energized*, *pleasant*, *happy*, *delight*, *liking*, *surprised*, *interest*, *absorbed*, *worried, shocked*, *dislike*, and *unsettled*. For the Aesthetic condition, our analysis identified five unique core emotions: *deeply moved*, *meaning*, *sentimental*, and *uncomfortable*, *scared*. For the Non-Aesthetic condition, we found two unique core emotions: *gripped* and *content*. For the Consumer condition, we found seven unique core emotions: *cheerful*, *curious*, *motivated, wonder*, *depressed*, *repelled*, and *irritated*. *perfection, astonished, engaged, enthusiastic, fascinated, agitated, unpleasant* overlapped across the Aesthetic and Non-Aesthetic networks, which point to degrees of engagement. *Beauty, touched, impressed, inspired, distaste,* and *oppressive* overlapped across the Aesthetic and Consumer networks, which include aesthetic valuation.

Overall, we find overlapping and distinct core emotions across the three conditions, highlighting the context-specificity of core emotions in aesthetic and non-aesthetic experiences.

## Discussion

Aesthetic emotions (AE) are emotions that arise when a person evaluates stimuli for their aesthetic appeal^17^. Whether these emotions are unique to aesthetic activities is contentious^10,11,88^. Our goal was to test the hypothesis that memories of aesthetic activities evoke a multidimensional emotional space that is organized differently than in other engaging activities. We did so by asking participants to recall aesthetic, non-aesthetic, or consumer activities, and then rate candidate AE evoked by these past activities. We then applied a computational psychometric network approach to represent and compare the space of these emotions across conditions. In line with current views on emotions being multidimensional and dynamic^18,19^, we focus on the set of emotions, or the emotional space that arises from different aesthetic and non-aesthetic activities. This contrasts with previous research on aesthetic emotions, which sometimes focus only on a single emotion (e.g.^89^).

Psychometric network analysis confirmed our primary hypothesis that the emotional space in recalling aesthetically engaging activities is organized differently compared to non-aesthetically engaging activities. At the macro-level, the Aesthetic network exhibited the highest clustering and lowest shortest paths and modularity compared to the two other networks. This pattern indicates that a wider range of emotions are activated in aesthetic recollections that “spread” from one emotion to the other more easily. This ease of spread suggests a more complex mixture of emotions that are also remembered more intensely as suggested by their higher Likert ratings. Nodes in the Non-Aesthetic network were more segregated from each other (with higher ASPL) and exhibited the highest modularity across the three networks. This pattern indicates that Non-Aesthetic activities elicit a more modular—being more compartmentalized into different sub-communities—pattern of emotions. More modular network structures inhibit the diffusion of activation across the network from one community to another, by getting “stuck” within a community^90–92^. Thus, non-aesthetic emotional memories are more focused, segregated, and compartmentalized. Finally, the Consumer network exhibited the lowest clustering and the highest ASPL, indicating the most segregated and clearly compartmentalized emotional space. Thus, the three networks, despite being comprised from the same nodes representing emotion terms, have distinct topologies. This finding supports our hypothesis that an aesthetic context organizes emotional spaces differently compared to other engaging contexts.

At the meso-level, the Aesthetic network clustered into five communities: (1) The Prototypical category include mixed emotions (*touched, moved, deeply moved, nostalgic, sentimental, longing, melancholic*) that give rise to deeper understanding (*insight*, *meaning*) and a re-organized sense of personal relevance (*humbled*, *inspired, motivated*). These mixed states are frequently theorized to be prototypical of aesthetic encounters and categorized together^17,20,36,38^; (2) Energizing emotions are positive and motivating (*perked up, enthusiastic, invigorated, spurred, energized, amused*). This cluster most closely matches the Animation factor observed in Schindler et al.^17^; (3) Pleasing, positive valanced emotions (*pleasant, content, delight, happy, cheerful, merry, humor,* etc.) of lower arousal than the Energizing emotions. This cluster includes common responses to nature (*calm, content, relaxed*) and great art (*grace, harmony, perfection, beauty*); (4) Captivation emotions comprising initial attentional capture (*surprised, astonished, impressed*) that lead to more sustained, cognitive and epistemic engagement (*liking, interest, curious, engaged, fascinated, stimulated, challenged*) and intense degrees of absorption and self-forgetting (*gripped, absorbed, enraptured, enchanted, lost myself, time flow*). Thus, this community includes the epistemic/contemplative emotions posited or observed as a discrete category by others^17,20,36^; (5) Negative valence terms associated with disliking, ranging from low to high arousal.

Schindler et al.^17^ organizes their 21 factors into four categories of aesthetic emotions: Prototypical, Pleasing, Epistemic, and Negative—but their factor analysis indicated 7 groups. Our data driven approach replicates their Negative, Prototypical, and Animation factors, but their Negative and Sadness factors form a single (Negative) community, and their Epistemic and Humor factors are subsumed by our Captivation and Pleasing emotion communities.

Overall, the community structure in all three networks show strong separation of positive and negative emotions, and in each condition, the negative clusters are rated low in recollection. Differences in community structures across the three conditions are also evident by visual inspection, revealing differences in the emotional landscape of each context. The distribution of color in Figs. 3 and 4 illustrates how the community structure from the Aesthetic condition fragments in the other two. For example, among the Prototypical emotions that cluster together in the Aesthetic condition are *baffled* and *overwhelmed*, suggesting these states of uncertainty may serve as vehicles to insight and feeling moved in aesthetic activities. However, in the Non-Aesthetic condition *baffled* and *overwhelmed* bridge Captivating and Negative emotions, suggesting a different role – mediating disengagement or greater absorption – in these contexts Similarly, *sentimental,* and *nostalgic* are more closely aligned with Pleasing emotions in non-Aesthetic activities. For the Consumer condition, *melancholic* and *overwhelmed* are unequivocally linked to negative emotions.

At the micro-level, the core analysis revealed some emotions that are common across these contexts, and others that vary by context. Common core emotions were *moved*, *energized*, *pleasant*, *happy*, *delight*, *liking*, *surprised*, *interest*, *absorbed*, *worried, shocked*, *dislike*, and *unsettled*. For the Aesthetic condition, we identified five unique core emotions: *deeply moved*, *uncomfortable*, *sentimental*, *meaning*, and *scared*^17,20,36^. The overlap between Aesthetic and Non-Aesthetic core items suggests the importance of engagement *(perfection, astonished, engaged, enthusiastic, fascinated, agitated, unpleasant),* and for Aesthetic and the Consumer condition, consistent with our intuitions*,* the overlap indicate valuation in both (*beauty, touched, impressed, inspired, distaste, and oppressive)*.

Our results confirmed the relevance of commonly used measures of aesthetic appreciation (*beauty, liking, interest*) and emotions considered characteristic (*moved, deeply moved, touched, sentimental, fascination*^17,20,36^). By contrast, three typically considered AE did not emerge as core – *wonder, sublime,* and *awe*. For *sublime* and *awe*, their peripheral status might arise because these intense emotions only occur in rare peak experiences. Our method identifies core emotions as those consistently recalled by participants. Rarified emotions such as awe, even if important, would not appear as core in this analysis. Our observation of *wonder* might reflect a translation issue rather than the emotion being of low relevance.

From general assessments, aesthetic activities were recalled with greater emotional intensity and were least vividly recalled. This observation suggests that Aesthetic memories are based less on sensorial and more on emotional aspects than the other two conditions. Consistent with this interpretation, all emotion terms except negative ones were rated higher in Aesthetic than other conditions. Consumer recollections were the least emotionally intense memories. These observations are perhaps in keeping with the instrumental nature of consumer engagements, which might not have the same emotional residue as non-instrumental activities.

Finally, while the Aesthetic network was most similar to the Non-Aesthetic network at a macro level, its meso-level community structure was most similar to the Consumer network. Further research is needed to understand the dissociation of the similarity across networks in these levels. However, network analysis is typically conducted independently across network scales, as each scale relates to different aspects of the complex system it represents^93^. At the community level, the similarity of Aesthetic and Consumer networks perhaps occurred because both draw on aesthetic valuation despite the Consumer condition being instrumental. This emotional similarity, albeit different in intensity, supports a view of consumer behavior as a form of everyday aesthetics^94,95^.

Going back to the original question motivating our research, are there such a thing as AE? While Menninghaus argues that AE exist, defined as evaluations of subjective perceived aesthetic virtues^10,88^, Skov and Nadal strongly argue against the existence of AE^11^. We propose an intermediate position, by demonstrating that context affects the organization of a multi-dimensional emotional space.

### Limitations

First, the AESTHEMOS was developed in German, which has unique linguistic properties compared to English. While the AESTHEMOS was translated it into English, linguistic nuances may add noise to the assessment of aesthetic emotions in English. For example, the AESTHEMOS includes two items, one related to being *moved* and another to being *deeply moved*. While in English these two items would likely be merged to one item, they are separated in German.

Second, our manipulation is based on past recollection. Likely there is a difference in the emotional space activated when people are experiencing vs. recalling events^96^. Future research could extend our findings by measuring emotions in real-time compared to past recollection.

Finally, we did not test for a broad set of emotions derived more generally. By restricting our analysis to postulated AEs, rather than a general catalog of emotion terms, our approach is a narrowly focused test of the hypothesis that distinctions between aesthetic and non-aesthetic emotions exist. If such distinctions do not exist, then these purportedly AE would apply similarly to other emotion evoking recollections. Future design could start with a larger set of emotions that may be relevant to other (non-aesthetic and consumer) contexts. Additionally, one could build on a differential weighting approach to query emotional spaces specific to different art forms, such as visual art versus music. Similarly, studies in other cultures or languages might reveal subtle differences. Just as different languages categorize color or space differently, aesthetic emotions are likely to be in part culturally mediated constructs because of differences in how encounters are interpreted and how language is deployed. Aesthetic emotion words without English translation (e.g. *wabi sabi* in Japanese; *schandenfreude* in German; *soldades* in Portuguese) illustrate alternative delineations of the phenomenology of affect.

## Conclusions

By comparing the memories of a multi-dimensional emotion space of purported AE with engaging activities, we demonstrate that relevant emotional patterns attributed to these activities depend on their context. Aesthetic emotions constitute a range of human emotions that are weighted differently in aesthetic experiences compared to other encounters.

In accordance with the context-specific position that we promote, Menninghaus et al. acknowledge that “linguistic terms used to designate emotions that in their predominant meaning have no necessary bearing on aesthetic evaluation can acquire such an implication by virtue of a context-driven meaning activation”^88^; p. 651. Do aesthetic emotions exist? Aesthetic emotions are not a unique *set* of emotions, rather they are emotions with a distinct *organization* in aesthetic activities.

## Data Availability

Data of this study is available on the Open Science Forum repository at https://osf.io/jwdzf/?view_only=1a8e8b6f96ab457aa94ffab2f7451842.

## Appendix 1

### Aesthetic condition

This is a study about experiences that engage our attention. Please think of a specific and vivid memory of a time when you were deeply engaged in listening to or looking at some kind of art – e.g. reading a novel, watching a film, listening to music, attending a ballet, looking at a painting, beholding architectural interior or exteriors, etc. Take a minute to recall the experience in as much detail as you can – the art, where you were, who you were with, how you felt, what you thought, etc.

Please describe the experience and what you were thinking and feeling, with as much specificity as possible.

### Non-aesthetic condition

This is a study about experiences that engage our attention. Please think of a specific and vivid memory of a time when you were deeply engaged in listening to or looking at some kind of activity – e.g. reading the news, watching a political debate, listening to a lecture, attending a sporting event, witnessing an argument, studying a map, etc. Take a minute to recall the experience in as much detail as you can – the activity, where you were, who you were with, how you felt, what you thought, etc.

Please describe the experience and what you were thinking and feeling, with as much specificity as possible.

### Consumer condition

This is a study about experiences that engage our attention. Please think of a specific and vivid memory of a time when you were deeply engaged in purchasing some kind of product in a store – e.g. buying a book, a phone, clothing, groceries, furnishings or decor, etc. Take a minute to recall the experience in as much detail as you can – the activity, where you were, who you were with, how you felt, what you thought, etc.

Please describe the experience and what you were thinking and feeling, with as much specificity as possible.

## Appendix 2

We would now like to ask you a few questions on how you felt during the experience you just described. You will be presented with several different statements. For each statement, please choose the response that best matches your personal experience. For each statement, the responses will range from *not at all* to *very much*. Please only indicate how you actually felt. If that statement does not represent a feeling you had during this experience, please choose *not at all*.

## Appendix 3

**Table:**

#	Item	Node label	Aesthetic core	Non-Aesthetic core	Consumer core	Aesthetic communities	Non-Aesthetic communities	Consumer communities
1	Filled me with longing	Longing	0	0	0	1	1	1
2	Spurred me on	Spurred	0	0	0	2	1	2
3	Invigorated me	Invigorated	0	0	0	2	2	2
4	Made me cheerful	Cheerful	0	0	1	3	2	3
5	Felt oppressive	Oppressed	1	0	1	4	3	4
6	Gripped me	Gripped	0	1	0	5	1	5
7	Calmed me	Calm	0	0	0	3	2	3
8	Made me angry	Anger	0	1	1	4	3	6
9	I found it sublime	Sublime	0	0	0	5	2	3
10	Made me feel content	Content	0	1	0	3	2	3
11	Made me happy	Happy	1	1	1	3	2	3
12	Touched me	Touched	1	0	1	1	1	5
13	Delighted me	Delight	1	1	1	3	2	3
14	I found it perfect	Perfection	1	1	0	3	2	3
15	Felt deeply moved	Deeply moved	1	0	0	1	1	5
16	Worried me	Worried	1	1	1	4	3	6
17	Challenged me intellectually	Challenged	0	0	0	5	1	2
18	Was not aware of myself	Lost myself	0	0	0	5	1	4
19	Was impressed	Impressed	1	0	1	5	2	1
20	Made me feel melancholic	Melancholic	0	0	0	1	1	6
21	Made me feel uncomfortable	Uncomfortable	1	0	0	4	3	6
22	Felt depressed	Depressed	0	0	1	4	3	6
23	Felt a sudden insight	Insight	0	0	0	1	1	2
24	Was mentally engaged	Engaged	1	1	0	5	1	2
25	Liked it	Liking	1	1	1	5	2	3
26	Was shocking to me	Shocked	1	1	1	4	1	4
27	Repelled me	Repelled	0	0	1	4	3	4
28	I found it beautiful	Beauty	1	0	1	3	2	1
29	Was attracted	Attracted	0	0	0	3	2	3
30	Made me feel enthusiastic	Enthusiastic	1	1	0	2	2	3
31	Baffled me	Baffled	0	1	1	1	1	4
32	Felt humbled	Humbled	0	0	0	1	1	1
33	Made me feel nostalgic	Nostalgic	0	0	0	1	2	5
34	Made me curious	Curious	0	0	1	5	1	2
35	Made me aggressive	Aggressive	0	0	0	4	3	4
36	Disliked it	Dislike	1	1	1	4	3	6
37	Felt something wonderful	Wonder	0	0	1	3	2	1
38	Tired me	Tired	0	0	0	4	3	6
39	Energized me	Energized	1	1	1	2	1	5
40	Irritated me	Irritated	0	0	1	4	3	6
41	Made me feel sentimental	Sentimental	1	0	0	1	2	5
42	Inspired me	Inspired	1	0	1	1	2	5
43	Made me merry	Merry	0	0	0	3	2	3
44	Surprised me	Surprised	1	1	1	5	1	2
45	Sensed a deeper meaning	Meaning	1	0	0	1	1	5
46	Sparked my interest	Interest	1	1	1	5	1	2
47	Agitated me	Agitated	1	1	0	4	3	6
48	I found it graceful	Grace	0	0	0	3	2	1
49	Was overwhelmed	Overwhelmed	0	0	0	1	1	6
50	Was enchanted	Enchanted	0	0	0	5	2	1
51	Felt awe	Awe	0	0	0	5	2	1
52	Perked me up	Perked up	0	0	0	2	2	3
53	Motivated me to act	Motivated	0	0	1	1	1	2
54	Felt absorbed in the experience	Absorbed	1	1	1	5	1	2
55	I found it pleasant	Pleasant	1	1	1	3	2	3
56	Scared me	Scared	1	0	0	4	3	6
57	Astonished me	Astonished	1	1	0	5	1	2
58	I found it ugly	Ugliness	0	0	0	4	3	6
59	Was funny to me	Humor	0	0	0	3	2	5
60	I found it unpleasant	Unpleasant	1	1	0	4	3	6
61	Felt confused	Confused	0	0	0	4	3	6
62	Felt that time was flying	Time flow	0	0	0	5	1	5
63	I found it distasteful	Distate	1	0	1	4	3	6
64	Felt indifferent	Indifferent	0	0	0	4	3	6
65	Was enraptured	Enraptured	0	0	0	5	1	3
66	Moved me	Moved	1	1	1	1	1	5
67	Made me sad	Sad	0	1	1	4	3	6
68	Bored me	Bored	0	0	0	3	3	4
69	Was unsettling to me	Unsettled	1	1	1	4	3	6
70	Put me in a dreamy mood	Dreamy	0	0	0	3	2	1
71	Amused me	Amused	0	0	0	2	2	5
72	I found it harmonious	Harmony	0	0	0	3	2	3
73	Stimulated my thoughts	Stimulated	0	1	1	5	1	2
74	Relaxed me	Relaxed	0	0	0	3	2	3
75	Fascinated me	Fascinated	1	1	0	5	1	1
